# 4-Borono-2-^18^F-fluoro-l-phenylalanine PET for boron neutron capture therapy-oriented diagnosis: overview of a quarter century of research

**DOI:** 10.1007/s12149-019-01347-8

**Published:** 2019-02-28

**Authors:** Kiichi Ishiwata

**Affiliations:** 1 Southern TOHOKU Drug Discovery and Cyclotron Research Center, Southern TOHOKU Research Institute for Neuroscience, 7-61-2 Yatsuyamada, Koriyama, 963-8052 Japan; 2 Department of Biofunctional Imaging, Fukushima Medical University, Fukushima, Japan

**Keywords:** ^18^F-FBPA, ^10^B-BPA, PET, BNCT, Malignant tumor

## Abstract

4-^10^B-Borono-2-^18^F-fluoro-l-phenylalanine (^18^F-FBPA) was developed for monitoring the pharmacokinetics of 4-^10^B-borono-l-phenylalanine (^10^B-BPA) used in boron neutron capture therapy (BNCT) with positron emission tomography (PET). The tumor-imaging potential of ^18^F-FBPA was demonstrated in various animal models. Accumulation of ^18^F-FBPA was higher in melanomas than in non-melanoma tumors in animal models and cell cultures. ^18^F-FBPA was incorporated into tumors mediated mainly by L-type amino acid transporters in in vitro and in vivo models. Tumoral distribution of ^18^F-FBPA was primarily related to the activity of DNA synthesis. ^18^F-FBPA is metabolically stable but is incorporated into melanogenesis non-enzymatically. These in vitro and in vivo characteristics of ^18^F-FBPA corresponded well to those of ^10^B-BPA. Nuclear magnetic resonance and other studies using non-radioactive ^19^F-^10/11^B-FBPA also contributed to characterization. The validity and reliability of ^18/19^F-FBPA as an in vivo probe of ^10^B-BPA were confirmed by comparison of the pharmacokinetics of ^18^F-FBPA and ^10^B-BPA and direct measurement of both ^18^F and ^10^B in tumors with various doses of both probes administered by different routes and methods. Clinically, based on the kinetic parameters of dynamic ^18^F-FBPA PET, the estimated ^10^B-concentrations in tumors with continuous ^10^B-BPA infusion were similar to those measured directly in surgical specimens. The significance of ^18^F-FBPA PET was verified for the estimation of ^10^B-concentration and planning of BNCT. Later ^18^F-FBPA PET has been involved in ^10^B-BPA BNCT of patients with intractable tumors such as malignant brain tumors, head and neck tumors, and melanoma. Usually a static PET scan is used for screening patients for BNCT, prediction of the distribution and accumulation of ^10^B-BPA, and evaluation of treatment after BNCT. In some clinical trials, a tumor-to-normal tissue ratio of ^18^F-FBPA > 2.5 was an inclusion criterion for BNCT. Apart from BNCT, ^18^F-FBPA was demonstrated to be a useful PET probe for tumor diagnosis in nuclear medicine: better tumor-to-normal brain contrast compared with ^11^C-methionine, differentiation of recurrent and radiation necrosis after radiotherapy, and melanoma-preferential uptake. Further progress in ^18^F-FBPA studies is expected for more elaborate evaluation of ^10^B-concentrations in tumors and normal tissues for successful ^10^B-BPA BNCT and for radiosynthesis of ^18^F-FBPA to enable higher ^18^F-activity amounts and higher molar activities.

## Introduction

Boron neutron capture therapy (BNCT) is a radiotherapeutic technique that selectively treats tumor cells with high-energy α particles and recoiling ^7^Li nuclei via ^10^B(n,α)^7^Li produced by the neutron irradiation of ^10^B-containing compounds located selectively in tumor tissues. The preferential characteristics of boron delivery agents for BNCT comprise high tumor uptake for several hours, relatively rapid clearance from blood and normal tissues, and low toxicity. So far, only two ^10^B-compounds have been used clinically, sodium ^10^B-borocaptate (Na_2_B_I2_H_11_SH or BSH) and 4-^10^B-borono-l-phenylalanine (^10^B-BPA), although a large number of ^10^B-compounds have been synthesized for over 50 years [1–4]. ^10^B-BPA is taken up by tumor tissues mainly by an L-type amino acid transporter (LAT), whereas BSH lacks such a tumor-specific uptake system and is taken into tumors by diffusion. To date, ^10^B-BPA appears to be in greater use in early clinical trials because of its very low toxicity and differential target/non-target extraction. It has been noted, however, that neither of these two agents, the so called second-generation boron-delivery agents, adequately fulfill the criteria required for BNCT and that third-generation agents are under development [2–4].

^10^B-BPA was originally synthesized as a possible ^10^B-boron-containing agent aimed at BNCT by Synder et al. [5]. Mishima et al. investigated ^10^B-compounds involved in melanogenesis, and applied ^10^B-BPA to a patient with malignant melanoma in BNCT for the first time [6]. Coderre et al. demonstrated that ^10^B-BPA could be applied to other tumors in experimental models: KHJJ murine mammary tumor, GS-9L rat glioma, and human U-87 MG glioma xenograft [7]. For successful BNCT in patients with malignant tumors, in vivo evaluation of the pharmacokinetics of ^10^B-BPA is one such problem that still needs to be resolved. 4-^10^B-Borono-2-^18^F-fluoro-l-phenylalanine (^18^F-FBPA) was developed in 1991 as a positron emission tomography (PET) probe for imaging and the evaluation of the pharmacokinetics of ^10^B-BPA in vivo [8]. After several characterization studies of ^18^F-FBPA using animal models in the early 1990s [8–12], ^18^F-FBPA PET has been clinically applied [13–19], and the significance of ^18^F-FBPA PET in BNCT using ^10^B-BPA has been established [1–4, 20–23]. However, ^18^F-FBPA PET has expanded to only limited numbers of PET facilities, mainly because BNCT is performed in a small number of institutes with nuclear reactors as a neutron source for BNCT. The problem of suitable reactors being located outside of hospitals makes clinical trials of BNCT very difficult. To overcome this problem, accelerator-based neutron sources for BNCT in the hospital were proposed [24], and the development of such accelerators is progressing [25]. Recently, a cyclotron that generates an epithermal-neutron source for BNCT was developed [23, 26, 27], and phase I and II clinical trials of BNCT using this cyclotron are progressing in Japan. Together with these sources of progress, further basic and clinical studies on ^18^F-FBPA aimed for BNCT have been reported in recent years.

Many reviews of BNCT including clinical studies of ^18^F-FBPA PET have been published; however, reviews focusing on ^18^F-FBPA PET, especially basic studies of ^18^F-FBPA, are limited in number [20–23]. Furthermore, in addition to BNCT, the usefulness of ^18^F-FBPA PET in the general diagnosis of tumors was also reported. This review summarizes basic and clinical studies on ^18^F-FBPA during the last quarter century, focusing on PET radiopharmaceutical science. Regarding basic research, the findings using non-radioactive ^19^F-FBPA and related ^10/11^B-BPA are also covered.

## Radiosynthesis of ^18^F-FBPA

**Fig. 1:**
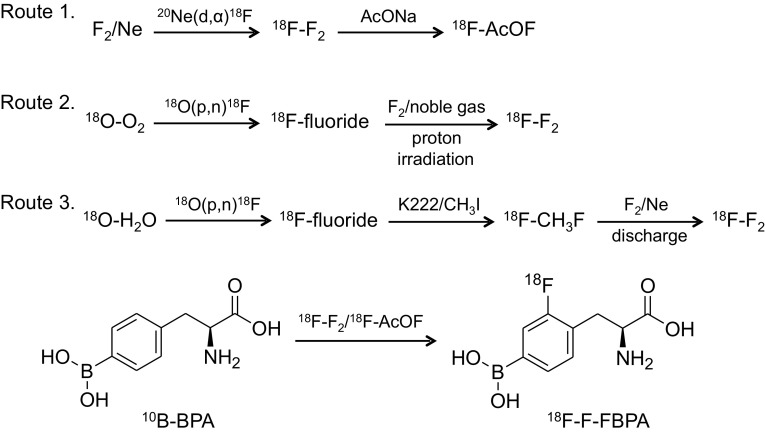
Radiosynthesis of 4-^10^B-borono-2-^18^F-fluoro-l-phenylalanine (^18^F-FBPA). The electrophilic substitution of ^10^B-BPA with ^18^F-acetylhypofluorite (^18^F-AcOF) and ^18^F–F_2_ produced ^18^F-FBPA as a predominant product, 4-^10^B-borono-3-^18^F-fluoro-l-phenylalanine as a minor product, and 2-, 3-, and 4-fluoro-l-phenylalanine as byproducts. ^18^F-AcOF is considered to have a higher selectivity compared to ^18^F–F_2_

^18^F-FBPA was synthesized by electrophilic substitution of ^10^B-BPA using carrier-added ^18^F–F_2_ produced via three routes (Fig. 1). First, carrier-added ^18^F–F_2_ was produced using the ^20^Ne(d,α)^18^F nuclear reaction and converted to ^18^F-acetylhypofluorite [8]. For this production an in-house cyclotron with a relatively large deuteron beam energy (about 8 MeV and more) is required, but the activity yields of ^18^F–F_2_ are limited. Consequently, the activity amount and molar activities of ^18^F-FBPA were very low: 440–1200 MBq and 20–130 MBq/µmol, respectively [8, 17, 28, 29]. It was noticed that the first synthesis was done by fluorination of racemic BPA [8]. Recently this route was re-examined in detail for the reliable production of ^18^F-FBPA for routine clinical use [30]. The formulation process of ^18^F-FBPA is available in the other syntheses, as described below. In this synthesis, the d-isomer of ^18^F-FBPA and contamination by trifluoroacetic acid used as a reaction solvent were confirmed to be negligible for the first time.

To obtain greater activity amounts of ^18^F–F_2_ and resulting relatively high molar activities, a second route for the production of ^18^F–F_2_ using the ^18^O(p,n)^18^F nuclear reaction with ^18^O–O_2_ [31] and ^18^O–H_2_O was devised [32]. This ^18^F–F_2_ can be produced using even a small proton-only accelerator. Thus, the activity yields of ^18^F-FBPA were improved greatly: 2000–5300 MBq [19, 33 (seemed to use BPA containing naturally abundant ^10/11^B-boron for ^18^F-FBPA synthesis)]. The third route using ^18^O–H_2_O-derived ^18^F–F_2_ achieved the highest molar activity 3700 MBq/µmol, but the activity yield of ^18^F-FBPA did not seem to be improved [34, 35].

^18^F-FBPA synthesis via the second and third routes may be referred to as the second-generation methods. Many people anticipate the feasibility of the third-generation synthesis of ^18^F-FBPA by nucleophilic fluorination using no-carrier-added ^18^F-fluoride produced using the ^18^O(p,n)^18^F nuclear reaction. The method could hopefully achieve a high activity amount and high molar activity of ^18^F-FBPA as the synthesis of ^18^F-FDG [36], but this has not yet been established.

## Experimental studies in in vitro and in vivo models

### Tumor accumulation of ^18^F-FBPA and PET imaging

In the 1990s, based on the clinical interest of Mishima and co-workers who applied ^10^B-BPA BNCT for patients with malignant melanoma [6], Ishiwata et al. demonstrated the potential of tumor accumulation of ^18^F-FBPA in mice with B16 melanoma and non-melanoma FM3A mammary carcinoma [9, 10] and in hamsters with Greene’s melanomas: melanotic no. 179 and amelanotic no. 178 [10, 11]. The accumulation of ^18^F-FBPA in these tumor models was evaluated using ex vivo γ-counting after tissue dissection. The ability of melanogenesis in tumors enhanced the uptake of ^18^F-FBPA. In a hamster model, melanotic no. 179 showed a 1.7-times higher uptake of ^18^F-FBPA than amelanotic no. 178, although both melanomas had similar metabolic activities when examined using a tracer uptake study with l-^14^C-methionine, 2-deoxy-d-^14^C-glucose and ^3^H-thymidine, which mainly reflect protein synthesis, glucose metabolism and DNA synthesis, respectively [10, 11]. In the mouse model, B16-F10 melanoma, which has a more highly metastatic potential and a more rapid growing rate but lower melanin content, showed lower uptake of ^18^F-FBPA than B16-F1 melanoma. These findings were explained by the fact that ^18^F-FBPA was partially incorporated in melanogenesis (see the section on the “Metabolism of ^18^F-FBPA”). Later, compared with non-melanoma cells, a higher uptake of ^18^F-FBPA in melanoma cells was also observed in vitro [37]. Prior these studies Coderre et al. reported a higher uptake of ^10^B-BPA in Harding–Passey melanoma than in the non-melanoma (mammary adenocarcinoma) in mice [38]. Ishiwata et al. used racemic ^18^F-FBPA in initial works [8–10], although it was known that the L-form of BPA was preferentially accumulated compared with the d-isomer [7]. Therefore, they further clarified that uptake of the L-form of ^18^F-FBPA was higher than that of the d-form in B16 melanoma [11]. Recently, similar findings were confirmed in C6 glioma-bearing rats [39].

The heterogeneous distribution of ^18^F-FBPA in tumor tissues was visualized ex vivo by whole-body autoradiography of mice with B16 melanoma or FM3A mammary carcinoma [9, 10]. Later, ^10^B-boron distribution in the tumors after injection of ^10^B-BPA was visualized ex vivo directly by neutron capture autoradiography using CR-39 nuclear track detectors [40].

In the 2000s, as technology advanced, PET imaging of ^18^F-FBPA in animal models was performed using high-spatial resolution PET scanners developed for small animals, although the first PET imaging was demonstrated in Greene’s melanoma-bearing hamsters with 8 mm of spatial resolution [11]. From a clinical interest in BNCT for patients with malignant brain tumors, Chen et al. performed a pilot study estimating the kinetic parameters of ^18^F-FBPA in F98-glioma-bearing rats using a PET apparatus with a spatial resolution of around 1.6 mm [41]. They measured quantitatively the forward (*K*_1_) and reverse transport rates (*k*_2_) of ^18^F-FBPA across the blood–brain barrier (BBB) and the anabolic (specific binding to the target) (*k*_3_) and reverse processes (*k*_4_) in tumor tissues based on a modified three-compartment physiological model [17, 18] (see the section on the “Assessment of ^10^B with ^18^F-FBPA-PET for l-BPA BNCT”). They found that tracer uptake capacity depends on *K*_1_, like in observed clinical studies [17, 18]. Later, it was confirmed that *K*_*1*_ is mediated mainly by LAT, especially LAT-1, in vitro and in vivo as described below [42, 43] (see the section on the “Transport of ^18^F-FBPA”).

Regarding in vivo imaging of FBPA, Porcari et al. reported that ^19^F magnetic resonance (MR) imaging of racemic ^10^B-enriched ^19^F-FBPA was an alternative technique to the monitoring of ^10^B-BPA [44]. Using a 7T MR-scanner they showed high accumulation of ^19^F-FBPA in C6 glioma in the rat brains, for which the images were superimposed on T2-weighted spin echo axial ^1^H images, after infusion of ^19^F-FBPA (300 mg/kg, no toxicity was confirmed) through the carotid artery. The signal-to-noise of each ^19^F MR image indicated the highest concentration of ^19^F at 2.5 h after infusion, and the concentration decreased gradually. Furthermore, quantitative ^19^F MR spectroscopic measurements of blood from the femoral vein show that the concentration of the total fluorinated compound decreases by approximately 22% from 1 to 2.5 h after infusion and then it remains constant until 4 h after infusion.

It is notable that MR imaging has been applied to other boron containing agents such as BSH [1, 45]. Both ^10^B and ^11^B are detectable by MR technique. ^11^B displays a higher sensitivity and better spectral resolution than ^10^B. However, the longer T_2_ relaxation time of ^10^B has a detection advantage. The possibility of the ^11^B MR imaging of BNCT agents in vivo in a dog and a patient with glioblastoma multiforme treated with BSH [46] and in a Fischer rat treated with Na_4_B_24_H_22_S_21_ has been documented [47]. Bendel et al. demonstrated the ^10^B MR imaging of ^10^B-enriched BSH in mice bearing M2R melanoma xenografts, and estimated boron concentration in kidney [48]. Further studies of MR imaging of boron agents including BPA are being conducted, but are beyond the scope of this review.

In the 2010’s several investigators using animal models bearing human cancer xenografts and high-spatial resolution PET scanners, visualized a heterogeneous distribution of ^18^F-FBPA in the tumor [40, 49], and performed further biological studies as described below.

### Cellular distribution of ^18^F-FBPA

The cellular distribution of ^18^F-FBPA in mice bearing two B16 melanoma sublines and FM3A mammary carcinoma was investigated using double-tracer microautoradiography [12]. The greatest amount of ^18^F-FBPA was observed in S phase melanocytes and the lowest amount was found in non-S phase non-melanocytes. ^18^F-FBPA accumulation was primarily related to the activity of DNA synthesis, as evaluated using [^3^H]thymidine, and secondarily to the degree of pigmentation in melanocytes. Prior to this study, Coderre et al. compared the tumoral distribution of ^10^B-BPA (neutron capture radiography) and ^3^H-thymidine (autoradiography) in Harding–Passey melanoma bearing mice. They found that the highest concentrations of l-BPA in the tumor corresponded closely with areas with high ^3^H-thymidine incorporation [38]. Notably, Bailey et al. found using flow cytometry that 24–72-h exposure to racemic FBPA increased the incorporation of bromodeoxyuridine into glioblastoma cells, suggesting that FBPA might directly affect the DNA synthetic pathway of tumor cells [50]. They considered that the previously reported increase of incorporation of FBPA in DNA synthesizing cells [12, 38] may actually reflect the action upon the DNA synthetic pathway of target cells.

Using human glioblastoma T98G cells, Chandra et al. visualized the cellular distribution of ^19^F-^10/11^B-FBPA prepared from l-BPA containing naturally abundant boron (80 atom% ^11^B, 20 atom% ^10^B) and ^10^B-BPA (> 95 atom% ^10^B) at the same dose using ion microscopy coupled with confocal laser scanning microscopy [secondary ion mass spectroscopy imaging] [51]. The mitochondria-rich perinuclear cytoplasmic region exhibited significantly lower ^19^F-fluorine and ^11^B-boron signals than the remaining cytoplasm and the nuclei, and ion microscopy observations revealed a nearly 1:1 distribution of ^19^F-fluorine and ^11^B-boron in subcellular compartments. This finding suggested that defluorination or decomposition of ^19^F-FBPA did not occur in tumor cells. No significant difference in the cellular localization of ^11^B-boron or ^10^B-boron was observed between the ^19^F-^10/11^B-FBPA and ^10^B-BPA.

### Tumor imaging potential of ^18^F-FBPA compared with other PET probes

The tumor imaging potential of ^18^F-FBPA was compared with other PET probes. Tumor uptake of racemic ^18^F-FBPA was similar to that of ^11^C-methionine in FM3A mammary carcinoma bearing mice [9]. In F98-glioma-bearing rats, tumor uptake of ^18^F-FBPA was lower than that of another artificial ^18^F-labeled amino acid derivative, *O*-2-^18^F-fluoroethyl-l-tyrosine, but the tumor-to-normal brain (*T*/*B*) ratios were rather higher [52]. Tumor uptake of ^18^F-FDG was much higher than that of these two ^18^F-labeled amino acids when dissecting tissues and measuring using γ-counter; however, the high uptake of ^18^F-FDG in the normal brain gave blurred brain tumor images. Watabe et al. compared ^18^F-FBPA, ^11^C-methionine, and ^18^F-FDG in a rat xenograft model of C6 glioma [42]. Tumor uptake values of ^18^F-FBPA and ^11^C-methionine were similar and lower than that of ^18^F-FDG, whereas the uptake values of ^18^F-FBPA and ^11^C-methionine in turpentine oil-induced inflammatory lesions were significantly lower than that of ^18^F-FDG. These findings suggested the usefulness of ^18^F-FBPA and ^11^C-methionine for differentiating between tumor and inflammation; however, differences in tumor-to-inflammatory lesion uptake ratios were not especially large in this model: ^18^F-FBPA, 1.7; ^11^C-methionine, 2.1; and ^18^F-FDG, 1.6.

### Transport of ^18^F-FBPA

Wittig et al. reported that ^10^B-BPA is transported in rat 9L gliosarcoma cells and Chinese hamster V79 cells by a LAT based on the findings for ^10^B-BPA import and efflux measurements in the presence of system L- and system A-specific substrates [53]. Among the system L family, particularly LAT-1, which is highly expressed in malignant tumors, may play a major role in the effectiveness of ^10^B-BPA in BNCT [54]. Wongthai et al. evaluated the subtype specificity. *K*_*m*_ values of ^10^B-BPA for ATB^0,+^, LAT-1 and LAT-2 (mainly expressed in normal tissues) were 137, 20, and 88 µM (LAT-2/LAT-1 = 4.3), respectively [55].

Regarding the transport of ^18^F-FBPA, Yoshimoto et al. found that ^18^F-FBPA was incorporated mainly into three human glioblastoma cell lines (74.5–81.1% of total uptake), by LAT [43]. ^18^F-FBPA uptake was decreased dose-dependently to 2.1–7.1% of control by 1 mM ^10^B-BPA. They also found that the contribution of LAT to ^14^C-methionine uptake was 48.3–59.4%, and suggested that ^11^C-methionine PET might overestimate the concentration of ^10^B-BPA in tumor tissues. Watabe et al. [42] also demonstrated that ^18^F-FBPA uptake was specific for LTA-1 in human embryonic kidney 293 cells (HEK293). *K*_*m*_ values of ^18^F-FBPA for LAT-1 and LAT-2 (normal cell type transporter) were 197 and 2810 µM (LAT-2/LAT-1 = 14), respectively. These findings suggested that ^18^F-FBPA is more specific for LAT-1 than ^10^B-BPA [55] and that ^18^F-FBPA is taken up less by tumor and normal tissues than ^10^B-BPA. The tumor-to-normal tissue (*T*/*N*) ratios of ^18^F-FBPA PET may be different from those of ^10^B-BPA in BNCT.

### Metabolism of ^18^F-FBPA

**Fig. 2:**
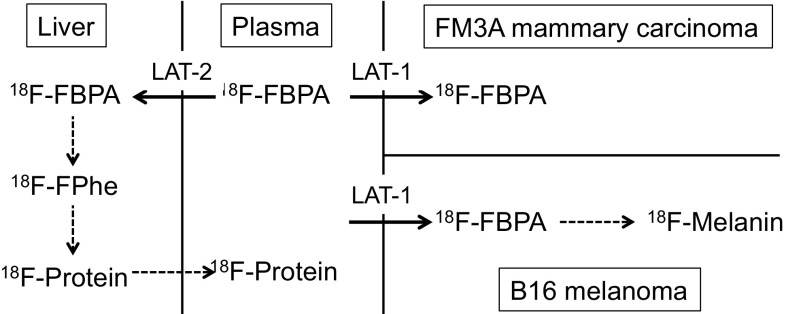
Metabolism of ^18^F-FBPA in C3H/He mice bearing FM3A mammary carcinoma and C57BL/6 mice bearing B16 melanoma. Transport of ^18^F-FBPA from plasma to the tumor and liver is mainly mediated by L-type amino acid transporter (LAT)-1 and − 2, respectively. Negligible protein-bound ^18^F-activity was found in FM3A mammary carcinoma [9]. ^18^F-2-Fluoro-l-phenylalanine (^18^F-FPhe) was not detected but speculated from an increasing protein-bound fraction of ^18^F-activity in plasma: 20% at 2 h in C3H/He mice [10] and 33% at 6 h in C57BL/6 mice [11]. The protein-bound fraction of ^18^F-activity (27% at 6 h) in B16 melanoma was evaluated as ^18^F-melanin [10]

Metabolic pathways of ^18^F-FBPA are summarized in Fig. 2 based on the results of early animal studies [9, 10]. In general, it is considered that the artificial amino acids are not incorporated into proteins. In non-melanoma FM3A mammary carcinoma, most ^18^F-activity was detected as ^18^F-FBPA over 6 h post injection, and the protein-binding fraction was negligible, whereas in B16 melanoma considerable amount of ^18^F-activity were detected in the protein-binding fraction (27% by 6 h), suggesting the involvement of ^18^F-FBPA in melanogenesis, as shown using double-tracer microautoradiography above [12]. On the other hand, the protein-binding fraction in plasma increased with time after injection of ^18^F-FBPA [9]. This finding suggested the deboronation of ^18^F-FBPA in vivo. In the liver, phenylalanine 4-monooxygenase may convert ^18^F-FBPA to 2-^18^F-fluoro-l-tyrosine, which was used in the synthesis of plasma proteins secreted into the blood stream [56]. Even if 2-^18^F-fluoro-l-tyrosine is re-circulated from the liver into the blood stream, it may make only a minor contribution to the total ^18^F-activity in tumor tissues. These findings suggested a slight discrepancy between the concentrations of ^18^F-activity and ^10^B in the animal studies to a certain extent, as described below. Regarding the metabolic change of BPA, Bendel et al. reported that the borate group was partly cleaved from BPA in the ^10^B MR analysis of human urine samples periodically collected from patients with head and neck squamous cell carcinoma [57].

No further studies on the metabolism of ^18^F-FBPA have been reported except for the work of Grunewald et al., in which plasma metabolites were analyzed for kinetic analysis of ^18^F-FBPA PET in tumor-bearing mice (> 96% unchanged ^18^F-FBPA in plasma at 60 min post injection) [58]. Clinically, three groups confirmed unchanged ^18^F-FBPA to be > 94% in plasma up to 50 min in dynamic ^18^F-FBPA PET [17, 34, 59], suggesting that ^18^F-FBPA is stable in humans at least as measured by PET.

### Validity and reliability of ^18^F-FBPA as a ^10^B-BPA probe

Several studies have indicated that FBPA and ^10^B-BPA exhibit similar pharmacokinetics and that ^18^F-FBPA can become an in vivo probe to monitor the behavior of ^10^B-BPA. Ishiwata et al. compared the ^10^B concentrations estimated by two methods in B16 melanoma-bearing mice and Greene’s melanoma-bearing hamsters after intravenous injection of a mixture of ^18^F-FBPA (1.0–2.6 mg/kg) and ^10^B-BPA (14–80 mg/kg) [11]. First, ^10^B derived from both ^18^F-FBPA and ^10^B-BPA was measured directly by inductively coupled plasma atomic emission spectroscopy (ICP–AES). Second, ^10^B was calculated from ^18^F-activity measured ex vivo using γ-counter and the molar activities that were determined as the ^18^F-activity per summed mass of ^18^F-FBPA and ^10^B-BPA based on the hypothesis that ^18^F-FBPA and ^10^B-BPA behave as the same compound in vivo. The ^10^B concentrations calculated from ^18^F-activity were comparable with those measured by ICP–AES in Greene’s melanoma but much lower in B16 melanoma. The discrepancy was larger in blood and muscle. The findings indicated that the ^18^F-FBPA was useful as an in vivo probe of ^10^B-BPA, although species or tissue differences exist to a certain degree between ^18^F signals and ^10^B concentrations.

Wang et al. showed that the time courses of ^10^B-BPA and ^18^F-FBPA measured ex vivo by ICP–MS and γ-counting, respectively, were similar in F98 glioma and normal brain with maximal levels at 1 h, when different doses of ^18^F-FBPA (0.5–0.8 mg/kg, estimated by the author) and ^10^B-BPA (172 mg/kg) were separately injected into two groups of F98 glioma-bearing rats [28]. However, a certain discrepancy was again observed between *T*/*B* ratios of ^10^B concentration in the two groups and between *T*/*B* ratios of ^10^B and those of ^18^F in rats given ^18^F-FBPA.

After these early studies, Hanaoka et al. performed dynamic whole-body PET/CT scans for 1 h in RGC6 glioma-bearing rats after intravenous injection of ^18^F-FBPA (1.7 mg/kg), and 1 h later the same rats were given intravenous injection of ^10^B-BPA (18.7 mg/kg), and found a significant positive correlation between the accumulation levels of ^18^F-FBPA and ^10^B-BPA measured ex vivo by ICP–MS in blood and in ten tissues including tumors in RGC6 glioma-bearing rats [29]. The group estimated the ^10^B concentrations using a practical formula [60]:

$$^{{10}}{\text{B}}\;{\text{ppm}}\,=\,0.0478\, \times \,1\;{}^{0}{\text{B-BPA}}\;{\text{dose}}\;\left( {{\text{mg}}/{\text{kg}}} \right) \times {}^{{18}}{\text{F}}\;{\text{SUV}}$$

SUV (standardized uptake value) is defined as (^18^F/ml tissue) × (g body weight/total injected ^18^F). However, this formula was determined to fit the ^18^F signal to ^10^B concentrations in the rat model, and cannot be applied to other species.

Watanabe et al. compared biodistributions of the same doses (500 mg/kg) of ^10^B-BPA and ^19^F-FBPA given separately in two groups of SCC-VII-bearing mice according to two administration protocols: subcutaneous injection and continuous subcutaneous infusion [61]. No difference between ^10^B-BPA and ^19^F-FBPA was noted in the time course of ^10^B measured by ICP–AES in tumor or normal tissues with the same administration protocol. However, the continuous-infusion group showed lower normal tissue-to-blood ratios of ^10^B than the subcutaneous injection group, whereas the ratios of tumor to brain, tongue, and muscle were larger in the continuous-infusion group than in the subcutaneous injection group.

The ^10^B concentration in the blood, rather than in normal tissue such as muscle, is often used as the reference to calculate the ^10^B concentration in tumors for BNCT [62]. Lin et al. found that the tumor-to-blood and tumor-to-muscle ratios of ^10^B (measured ex vivo by ICP–AES) were variable at the time of measurement, but the muscle-to-blood ratio of ^10^B remained constant about 1.31 at 30–45 min after intravenous injection of ^10^B-BPA (400 mg/kg) into mice bearing SAS human oral carcinoma xenografts [40]. They suggested that ^10^B concentrations in tumor and normal tissues be estimated using the normal tissue-to-blood ratio as a conversion factor (1.31 in this animal case). They recommended that before BNCT of patients the *T*/*N*, the tumor-to-blood, and normal tissue-to-blood ratios be determined using ^18^F-FBPA PET.

Grunewald et al. also indicated the equivalent tissue distribution patterns of different administration doses of ^18^F-FBPA (1.5 mg/kg) and ^10^B-BPA (200 mg/kg) in tumor-bearing mice [58]. The ^10^B concentration was analyzed ex vivo by prompt gamma activation analysis or quantitative neutron capture radiography. The organ-to-plasma ratios of ^10^B were well correlated with the organ-to-plasma ratios of ^18^F-activity measured ex vivo using γ-counter (*y* = 0.83*x* + 0.75; *r* = 0.93, *p* < 0.0001), and also well-correlated with the organ-to-heart ratios of ^18^F-activity measured in vivo by last PET frame (*y* = 0.76*x* + 0.28; *r* = 0.83, *p* = 0.0001). Because it is assumed that the heart ^18^F-activity represents mainly blood ^18^F-activity, the organ-to-plasma ratios of ^10^B could be estimated by the organ-to-heart (blood pool in left ventricle) ratios of ^18^F-activity measured in vivo PET.

Yoshimoto et al. compared the pharmacokinetics of ^18^F-FBPA (0.6–3.1 mg/kg, estimated by the author) by bolus intravenous injection and continuous intravenous 30-min infusion with/without ^10^B-BPA (250 mg/kg) using a mouse model bearing six human tumor xenografts [63]. All six tumors showed increasing uptake of ^18^F-FBPA after a bolus injection. SUVs in LN-229 human glioma at 50–60 min after three administration methods were similar: bolus injection 1.26, continuous infusion without ^10^B-BPA 1.22, and continuous infusion with ^10^B-BPA 1.12. In six tumors, a significant association was revealed between tumor uptake of ^18^F-FBPA by bolus injection and by continuous infusion (*r* = 0.92, *p* < 0.01). ^10^B-Boron concentration measured ex vivo by ICP–AES in tumors correlated with ^18^F-FBPA uptake regardless of the administration method.

### Enhancement of ^18^F-FBPA accumulation

For successful BPA BNCT high accumulation of ^10^B-BPA in tumors is essential. It has been reported that preloading with L-type amino acids such as l-tyrosine and l-DOPA enhances accumulation of l-BPA in malignant glioma and melanoma cells [48, 64–66]. When this approach was expanded to FBPA, similar enhancement phenomena were confirmed in the racemic ^10^B-enriched ^19^F-FBPA uptake in vitro in C6 glioma cells and in vivo in rats bearing C6 glioma by preloading of l-DOPA [67]. Additionally, ^18^F-FBPA uptake was enhanced in human and mouse tumor cell lines in vitro by preloading of l-DOPA, l-tyrosine, and l-BPA itself [37]. On the other hand, Grunewald et al. found that preloading of l-tyrosine, l-DOPA, and l-BPA did not increase the uptake of ^18^F-FBPA or ^10^B-BPA in any organs of mice bearing HuH-7 human hepatocellular carcinoma xenografts [58].

Regarding brain tumors, BBB disruption may be another approach to enhance the delivery of ^10^B-BPA [68–70]. In F98 glioma-bearing rats that received mannitol or cereport (a receptor-mediated permeabilizer-7), the tumor ^10^B concentrations after intracarotid injection of ^10^B-BPA were enhanced compared with controls, which resulted in significantly longer survival times after ^10^B-BPA BNCT. Using this animal model and a mannitol-induced hyperosmotic BBB disruption technique, Hsieh et al. confirmed enhanced tumor uptake and tumor-to-ipsilateral brain ratios of both ^10^B-BPA and ^18^F-FBPA after intracarotid injection [71]. They suggested that the pharmacokinetic parameter *k*_12_/*k*_21_ ratio (*k*_12_: rate constant of the central compartment to the peripheral compartment; *k*_21_: rate constant of the peripheral compartment to the central compartment) measured by ^18^F-FBPA PET may serve as a good indication for evaluating tumor uptake and tumor-to-brain ratio after intracarotid injection of ^10^B-BPA.

It is also known that pulsed high-intensity focused ultrasound (pulsed-HIFU) is able to disrupt the BBB to improve the delivery of macromolecules, such as antibodies and liposomal drugs. Wu et al. demonstrated that this technique enhanced tumor uptake of ^18^F-FBPA in mice bearing orthotopic SASC03 human tongue squamous carcinoma xenografts [49]. Immediately after pulsed-HIFU, tumor uptake of ^18^F-FBPA was 1.8 times that of the control at 60 min post injection; however, pulsed-HIFU did not affect the distribution of ^18^F-FBPA in most normal organs except the brain (3.1 times increase). The histology and expression of CD31 and Ki-67 were not changed by pulsed-HIFU. Yang et al. evaluated quantitatively the kinetics of ^18^F-FBPA in F98 glioma-bearing rats with pulsed-HIFU-induced BBB disruption [72], and found that the accumulation of ^18^F-FBPA in brain tumors and the tumor-to-contralateral brain ratio were significantly elevated. The *K*_1_/*k*_2_ ratio may be useful for indicating the degree of BBB disruption: *K*_1_ and *k*_2_ representing forward transport and reverse transport of ^18^F-FBPA across BBB.

## Clinical studies

### Radiation dosimetry

Before starting clinical studies on ^18^F-FBPA, radiation dosimetry was investigated in mice using racemic ^18^F-FBPA in a preclinical study [8]. Later, Ishiwata and co-workers evaluated the radiation dosimetry of l-enantiomer of ^18^F-FBPA in adult humans by dynamic whole-body PET scanning, and indicated that the effective dose of ^18^F-FBPA (23.9 µSv/MBq, *n* = 6) in humans was similar to that of other ^18^F-fluorinated PET probes such as ^18^F-FDG, *O*-(2-^18^F-fluoroethyl)-l-tyrosine, and 6-^18^F-fluoro-l-dopa (19–29, 16.5, 19.9 µSv/MBq, respectively) [73]. Kono et al. also reported slightly smaller effective doses of ^18^F-FBPA compared with ^18^F-FDG in adult and pediatric patients [74]. The effective dose of ^18^F-FBPA in pediatric patients (31 µSv/MBq, *n* = 3) was larger than that in adult patients (15 µSv/MBq, *n* = 6).

### Assessment of ^10^B with ^18^F-FBPA-PET for l-BPA BNCT

**Fig. 3:**
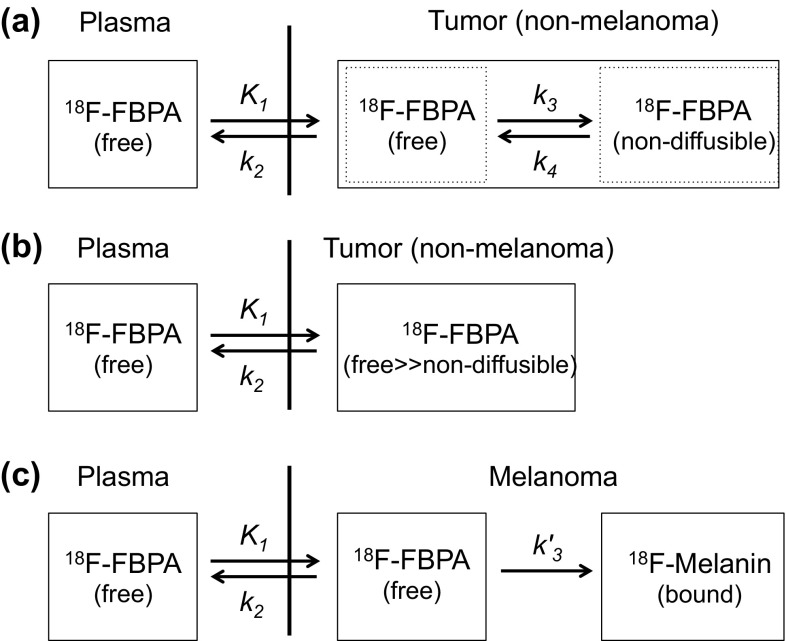
Kinetic models of ^18^F-FBPA. **a** Reversible two-tissue compartmental model. The model proposed by Imahori et al. was simplified [17]. **b** One-tissue compartmental model. The model has a very low or negligible non-diffusible component (retention process). **c** Irreversible two-tissue compartmental model. The possible model for melanoma based on the incorporation of ^18^F-FBPA into melanogenesis [10, 11]

Imahori, Mishima, and collaborators performed initial clinical trials of ^18^F-FBPA PET in patients with malignant brain tumors [13–15] and metastatic melanomas [16]. They performed dynamic PET scans with arterial blood sampling after intravenous injection of ^18^F-FBPA. First, Imahori et al. calculated the utilization ratio (integration of the ^18^F-activity that appears in arterial blood relative to the total injection dose) and incorporation constant (the amount of incorporated ^18^F-FBPA in the tumor tissue divided by plasma ^18^F-FBPA integrated over time) of ^18^F-FBPA, and estimated ^10^B-boron concentrations. The values estimated were generally higher but were very close to those measured ex vivo in surgical specimens of patients after ^10^B-BPA infusion by the ICP–AES [15]. To improve the ^10^B estimation method, Imahori et al. determined kinetic parameters (*K*_1_, *k*_2_, *k*_3_, and *k*_4_) based on a three-compartment model (Fig. 3a) [17, 18]. *K*_1_, which is mainly mediated by LAT-1 as described above, was a major factor determining the accumulation of ^18^F-FBPA, but *k*_*3*_ did not correlate with the degree of malignancy [17]. Subsequently, in seven patients with continuous infusion of ^10^B-BPA in a way similar to that used in the clinical practice of BNCT, they estimated ^10^B concentrations in tumors by the segmental convolution method using these rate constants, compared with those in the surgical specimens, and verified the ^18^F-FBPA PET method to estimate ^10^B concentrations [18]. Similarly, Kabalka et al. further extended this model to a four-compartment model for ^18^F-FBPA PET, and determined the optimal irradiation window for effective ^10^B-BPA BNCT from the calculated tissue ^18^F-activity based on a simulated continuous infusion of ^18^F-FBPA using kinetic parameters [19].

It is notable that the compartment analyses by Imahori et al. and Kabalka et al. included the retention process of ^18^F-FBPA or non-diffusible ^18^F-FBPA (expressed as *k*_3_ and *k*_4_) in malignant brain tumors in spite of the metabolic stability of ^18^F-FBPA because of the increasing time–activity curves for about 40 min followed by a very slow decrease. On the other hand, Havu-Aurén et al. reported that benign neoplasms showed an initial uptake of ^18^F-FBPA at 3–5 min post injection followed by a gradual decrease, indicating very low or negligible retention processes (corresponding to the model in Fig. 3b), and that *K*_1_ was higher than *k*_3_, suggesting that transport rather than metabolism governed the uptake of ^18^F-FBPA [35]. They described that some of these benign neoplasms might be amenable to BNCT based on the results of ^18^F-FBPA PET. In our experience, many tumors in the head and neck showed similar time–activity curves without the retention processes, like in benign neoplasms. Only in malignant melanomas, free ^18^F-FBPA may be incorporated into melanin by non-enzymatic polymerization in melanogenesis (Fig. 3c), as suggested in metabolite analysis of ^18^F-FBPA in melanoma-bearing animal models [10, 11]. In a kinetic analysis of *O*-2-^18^F-fluoroethyl-l-tyrosine with characteristics similar to those of ^18^F-FBPA [75–77], it was best modeled by a reversible two-tissue compartmental model rather than a one-tissue compartmental model or irreversible two-tissue compartment model [77].

These dynamic ^18^F-FBPA PET studies were a practical and clinically useful method as a prognostic and therapeutic indicator of malignant brain tumors [78], and were applied to planning BNCT for malignant brain tumors [79] and malignant melanoma [80]. In 22 of 98 glioma patients in Finland who received BNCT, from 1999 to 2011, a kinetic model based on ^18^F-FBPA PET predicted + 11% and + 36% higher total weighted doses delivered to tumor and normal brain, respectively, than previously estimated doses due to the non-constant tumor-to-blood concentration ratios [81].

### Practical use of [^18^F]FBPA PET for BNCT

**Fig. 4:**
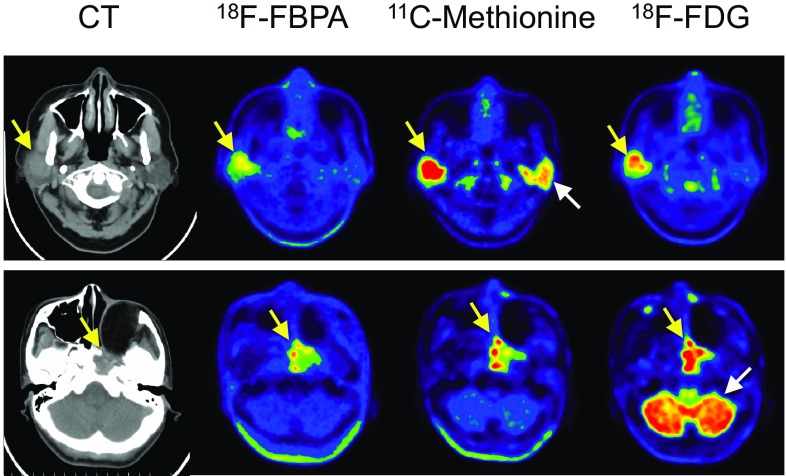
^18^F-FBPA, ^11^C-methionine, and ^18^F-FDG PET/CT images of patients with salivary gland duct carcinoma (upper row) and squamous cell carcinoma (lower row). Three PET scans were performed during a 2-week interval in the Southern TOHOKU Research Institute for Neuroscience (unpublished data, approved by the institutional ethics committee #224). The PET/CT scanner used was Discovery PET/CT 610 (GE Healthcare, Milwaukee, WI), and the injected radioactivity doses of ^18^F-FBPA, ^11^C-methionine, and ^18^F-FDG (upper low and lower row) were 4.3 and 4.6, 6.7 and 7.4, and 3.6 and 3.6 MBq/kg body weight, respectively. Yellow arrows; carcinomas; white arrows, salivary gland (upper row) and cerebellum (lower row). PET images are shown in the scale of SUV 0–6 except for ^18^F-FDG in the lower row (SUV 0–10). SUV_max_ values of ^18^F-FBPA (40–60 min post injection), ^11^C-methionine (20–30 min post injection), and ^18^F-FDG (50–60 min post injection) were 3.6, 9.7, and 7.1, respectively, in the upper row, and 5.9, 7.2, and 18.5, respectively, in the lower row

^18^F-FBPA PET before BNCT is useful for patient selection and prediction of the distribution and accumulation of ^10^B-BPA, and follow-up ^18^F-FBPA PET after BNCT is helpful to evaluate therapeutic effect. For effective BNCT accumulation of a large amount of ^10^B atoms, approximately 10^9^ atoms of ^10^B per cell or 20–35 µg ^10^B/g, in tumor cells are required, and at the same time a high *T*/*N*^10^B concentration ratio of greater than 1 and preferably 3–5 is required to ensure a therapeutic dose to the tumor with a minimal background radiation dose [82, 83]. From a practical point of view, there is an easy approach for ^18^F-FBPA PET for screening of patients suitable for BNCT. To evaluate the *T*/*N* ratio of the ^10^B concentration being over 3.0 pre-BNCT, it may be sufficient to compare the *T*/*N* ratio of ^18^F-FBPA uptake obtained by an appropriate static scan of ^18^F-FBPA PET. Nariai et al. indicated that in patients with malignant brain tumors the *T*/*B* ratio of ^18^F-FBPA after a bolus injection of ^18^F-FBPA had a significant linear correlation with the *T*/*B* ratio of ^10^B estimated by 1-h constant infusion of ^10^B-BPA, as simulated using the Runge–Kutta algorithm [84]. This type of quantitative evaluation has not been tried in other malignant tumors; however, Morita et al. recently reported that the *T*/*N* ratios of ^18^F-FBPA in head and neck cancers and malignant melanoma were not significantly changed over 120 min in spite of a slight decrease in ^18^F-FBPA uptake [85]. In evaluating the *T*/*N* ratio of ^18^F-FBPA in patients with head and neck cancers, it is notable that the uptake of ^18^F-FBPA was higher in the dorsum tongue, submandibular gland, parotid gland, and tongue in this order among normal tissues in the oral and maxillofacial regions than in normal brain [86]. The averaged ratios (*n* = 8) of *T*/*B* and tumor-to-dorsum tongue were 3.25 (range 2.34–5.40) and 1.25 (range 0.95–2.10), respectively. The ratios varied depending on the location of the tumor, type of tumor, and scan time post injection. For example, in Fig. 4a, tumor (salivary gland duct carcinoma)-to-surrounding normal tissue ratios was 3.2, whereas the tumor-to-contralateral normal salivary gland ratio was 2.3 (unpublished data). In an early study, Kabalka et al. indicated that the lung and peri-oral mucous gland showed intense ^18^F-FBPA activity [80].

To predict the accumulation (concentration) of ^10^B-BPA in tumors, first dynamic and quantitative ^18^F-FBPA PET was performed as described above. On the contrary, in BNCT the ^10^B concentration in the blood is often used as the reference to calculate the ^10^B concentration in tumors [62]. Therefore, in the practical use of ^18^F-FBPA PET for ^10^B-BPA BNCT, the ^10^B concentration in the tumor has been estimated by multiplying the ^10^B concentration in blood measured during neutron irradiation by the tumor-to-blood ratio of ^18^F-FBPA PET [87]. For this purpose, Isohashi et al. proposed the use of the image-derived tumor-to-blood ratio of ^18^F-FBPA [59]. They used ^18^F-activity in the left ventricle instead of ^18^F-activity in the blood, with correction for underestimation due to the partial volume effect and reduction of ^18^F-activity in the blood.

Estimating the ^10^B concentration by relative tumor uptake of ^18^F-FBPA against normal tissue and plasma has a certain practical benefit but does not predict possible adverse effects on the surrounding normal tissue by neutron irradiation. To avoid these risks the ^10^B concentration of the normal tissue should be estimated correctly. Recently, Shimosegawa et al. proposed a method to evaluate ^10^B-BPA accumulation directly in normal organs by ^18^F-FBPA PET before BNCT [88]. They assumed boldly that ^10^B-BPA and ^18^F-FBPA behave in the body as the same compound even after different injection doses, and they calculated the ^10^B concentrations in organs by multiplying the relative accumulation of ^18^F-FBPA per g tissue by the therapeutic dose (g) of l-BPA. At present, this method has not been validated in BNCT.

Thus far, ^18^F-FBPA PET has been utilized in ^10^B-BPA BNCT for patients with malignant brain tumors, recurrent tumors of the head and neck region, and malignant melanomas by many investigators (see reviews, [1, 2, 21–23, 89]). ^18^F-FBPA PET was a part of the inclusion criteria for BNCT clinical phase I/II trials of the treatment of recurrent head and neck cancers in Finland [90] and in Taiwan [91, 92]. In the Finnish study, ^18^F-FBPA PET was performed in 15 of 30 patients before BNCT and then in 7 patients after BNCT to evaluate treatment response. In the Taiwanese study, in 17 patients the *T*/*N* ratio of ^18^F-FBPA was > 2.5 as an inclusion criterion for one- or two-fractions of BNCT, and the treatment response was evaluated again by ^18^F-FBPA PET.

As shown in the Finnish study, ^18^F-FBPA PET post BNCT is involved to evaluate the treatment response in the typical BNCT protocol. For this purpose, ^11^C-methionine PET was equally useful as ^18^F-FBPA PET [93, 94]. ^18^F-FDG PET, which is generally used to evaluate the efficacy of treatment of tumors, was also used successfully to evaluate maxillary sinus cancer invading into the orbital fossa after BNCT [95]; however, it should be noted that ^18^F-FDG accumulates in the inflammatory tissue post radiotherapy and normal brain.

### Applicability of other PET probes for BNCT

If PET diagnosis with more common probes such as ^18^F-FDG and ^11^C/^18^F-amino acids is applicable to the screening of patients to assess their suitability for BNCT, BNCT can be performed effectively. Nariai et al. performed PET with ^18^F-FBPA and ^11^C-methionine in 12 patients with malignant glioma on the same day, and found that the estimated *T*/*B* ratio of ^10^B after a 1-h constant infusion of ^10^B-BPA and *T*/*B* ratios of ^18^F-FBPA and ^11^C-methionine on static conditions showed significant linear correlations [84]. Yamamoto et al. used both ^18^F-FBPA and ^11^C-methionine to evaluate ^10^B-BPA uptake in a BNCT trial of glioblastoma [96]. Watanabe et al. also compared ^18^F-FBPA and ^11^C-methionine PET in seven patients with head and neck tumors with intervals of less than 3 weeks in six cases except for a 123-day interval in one case [97]. They also described that ^11^C-methionine PET might be used instead of ^18^F-FBPA PET to select candidates for BNCT. However, ^11^C-methionine was taken up at higher levels than ^18^F-FBPA by tumors, as well as many normal tissues as a natural amino acid, and therefore, the *T*/*N* ratios would not be suitable for evaluating tumor accumulation in some normal organs such as the submandibular gland, liver, heart, stomach, pancreas, spleen, and bone marrow.

Kurihara et al. also compared ^18^F-FBPA and ^18^F-FDG PET in 20 patients with head and neck cancers [98, 99]. A significant correlation was observed between maximal SUVs (SUV_max_) of ^18^F-FBPA (4.13, *n* = 20) and ^18^F-FDG (9.40, *n* = 20), and an SUV_max_ of ^18^F-FDG ≥ 5.0 is considered to correspond to a *T*/*N* ratio of ^18^F-FBPA ≥ 2.5 as the threshold value for prediction of BNCT [98].

Figure 4 shows representative images of ^18^F-FBPA, ^11^C-methionine, and ^18^F-FDG PET (unpublished data). The characteristics of the three probes are clearly demonstrated: a lower tumor uptake of ^18^F-FBPA compared with ^11^C-methionine and ^18^F-FDG and a high uptake of ^11^C-methionine in the normal salivary glands.

### General use of ^18^F-FBPA in PET diagnosis

In general, ^11^C/^18^F-amino acids such as ^11^C-methionine and *O*-2-^18^F-fluoroethyl-l-tyrosine are useful for the diagnosis of brain tumors, and ^18^F-FBPA is also useful, as described above. A benefit of artificial amino acids such as *O*-2-^18^F-fluoroethyl-l-tyrosine and ^18^F-FBPA is their very low uptake in the normal brain due to rapid clearance without metabolic alteration, although ^11^C-methionine accumulates in the normal brain to a certain degree due to the metabolic pathways of methionine. Consequently, it is considered that artificial amino acids give higher contrast tumor images than ^11^C-methionine. This is true when the *T*/*B* ratio of ^18^F-FBPA was compared to that of ^11^C-methionine in 12 patients with glioma on the same day [84]. The ratio of ^18^F-FBPA was 1.61-fold higher than that of ^11^C-methionine, although the SUV of ^18^F-FBPA was 0.87-fold lower than that of ^11^C-methionine (personal communication).

Miyatake and colleagues reported that ^18^F-FBPA PET is useful for differential diagnosis among radiation necrosis, pseudo-progression, and progression after BNCT for malignant brain tumors [100–103]. They further described that both ^18^F-FBPA and ^11^C-methionine PET are equally useful for this purpose [94]. Prior to these studies, the usefulness of ^11^C-methionine PET for the differentiation of recurrent and radiation necrosis after stereotactic radiosurgery of malignant glioma was reported [104]. Recently Beshr et al. confirmed the possibility of ^18^F-FBPA PET for the differentiation of recurrence from radiation necrosis in 12 patients with irradiated brain tumors. The four parameters investigated were mean SUV, SUV_max_, metabolic tumor volume, and total lesion uptake, and their ratios of recurrence to radiation necrosis were 2.5, 4.2, 3.8, and 9.8, respectively [105].

Differential diagnosis between inflammatory lesions and tumors by ^18^F-FBPA PET was suggested from an animal study [42]; however, no clinical studies have been reported to date.

As described in section “Tumor accumulation of ^18^F-FBPA and PET imaging”, melanomas in animal models showed higher uptake of ^18^F-FBPA than non-melanoma tumors, probably due to the trapping mechanism of ^18^F-FBPA in melanogenesis. This melanoma-preferential uptake of ^18^F-FBPA was confirmed clinically by Morita et al. [85]. ^18^F-FBPA showed a slight washout pattern in squamous cell carcinoma of the head and neck (*n* = 20) and a persistent pattern in malignant melanoma (*n* = 8). The melanomas-to-squamous cell carcinoma uptake ratios of ^18^F-FBPA increased slightly (1.69, 1.73, and 1.93) with time post-injection (30, 60, and 120 min, respectively).

## Concluding remarks

^18^F-FBPA has been developed for monitoring the pharmacokinetics of ^10^B-BPA used in BNCT and characterized as a ^10^B-BPA probe in basic studies in vitro and in vivo of tumor imaging potential, transport, cellular distribution, metabolism, validity, and reliability. Tumor uptake of ^18^F-FBPA depends mainly on transport by LAT. Tumoral distribution of ^18^F-FBPA was primarily related to the activities of DNA synthesis and melanogenesis. Pharmacokinetics of ^18^F-FBPA reflects mostly that of BPA, although there are slight discrepancies between the two compounds. Clinically, kinetic analysis based on ^18^F-FBPA PET has successfully estimated the ^10^B-concentration in tumor tissues, and the significance of ^18^F-FBPA PET in ^10^B-BPA BNCT has been established. ^18^F-FBPA PET has been involved in the practical ^10^B-BPA BNCT for the treatment of patients with intractable tumors and used for screening of patients, prediction of the distribution and accumulation of ^10^B-BPA, and the evaluation of treatment. Static ^18^F-FBPA PET scans are generally used to measure the *T*/*N* and tumor-to-blood ratios of ^18^F-FBPA. However, it has not necessarily been clarified whether PET findings predict the prognosis of BNCT. Furthermore, recent studies have suggested that ^18^F-FBPA PET could be used more elaborately for successful ^10^B-BPA BNCT. Apart from BNCT, ^18^F-FBPA is considered to be a useful PET probe for tumor diagnosis; however, for general diagnosis in nuclear medicine, the third-generation synthesis of ^18^F-FBPA by nucleophilic fluorination using ^18^F-fluoride that will enable higher activity amounts and higher molar activities, is required.

## Abbreviations

BBB: Blood–brain barrier
BNCT: Boron neutron capture therapy
^10^B-BPA: 4-^10^B-Borono-l-phenylalanine
BSH: Sodium ^10^B-borocaptate
^18^F-FBPA: 4-^10^B-Borono-2-^18^F-fluoro-l-phenylalanine
ICP–AES: Inductively coupled plasma–atomic emission spectroscopy
ICP–MS: Inductively coupled plasma–mass spectroscopy
LAT: L-type amino acid transporter
MR: Magnetic resonance
PET: Positron emission tomography
Pulsed-HIFU: Pulsed high-intensity focused ultrasound
SUV: Standardized uptake value
*T*/*B*: Tumor-to-normal brain
*T*/*N*: Tumor-to-normal tissue

## References

[1] Wittig A, Michel J, Moss RL, Stecher-Rasmussen F, Arlinghaus HF, Bendel P (2008). Boron analysis and boron imaging in biological materials for boron neutron capture therapy (BNCT). Crit Rev Oncol Hematol.

[2] Barth RF, Vicente MGH, Harling OK, Kiger WS, Riley KJ, Binns PJ (2012). Current status of boron neutron capture therapy of high grade gliomas and recurrent head and neck cancer. Radiat Oncol.

[3] Moss RL (2014). Critical review, with an optimistic outlook, on boron neutron capture therapy (BNCT). Appl Radiat Isot.

[4] Barth RF, Mi P, Yang W (2018). Boron delivery agents for neutron capture therapy of cancer. Cancer Commun.

[5] Synder HR, Reedy AJ, Lennarz WMJ (1958). Synthesis of aromatic boronic acids. Aldehydo boronic acids and a boronic acid analog of tyrosine. J Am Soc Chem.

[6] Mishima Y, Honda C, Ichihashi M, Obara H, Hiratsuka J, Fukuda H (1989). Treatment of malignant melanoma by single thermal neutron capture therapy with melanoma-seeking ^10^B-compound. Lancet.

[7] Coderre JA, Glass JD, Fairchild RG, Micca PL, Fand I, Joel DD (1990). Selective delivery of boron by the melanin precursor analogue *p*-boronophenylalanine to tumors other than melanoma. Cancer Res.

[8] Ishiwata K, Ido T, Mejia AA, Ichihashi M, Mishima Y (1991). Synthesis and radiation dosimetry of 4-borono-2-[^18^F]fluoro-d,l-phenylalanine: a target compound for PET and boron neutron capture therapy. Appl Radiat Isot.

[9] Ishiwata K, Ido T, Kawamura M, Kubota K, Ichihashi M, Mishima Y (1991). 4-Borono-2-[^18^F]fluoro-d,l-phenylalanine as a target compound for boron neutron capture therapy: tumor imaging potential with positron emission tomography. Nucl Med Biol.

[10] Ishiwata K, Ido T, Honda C, Kawamura M, Ichihashi M, Mishima Y (1992). 4-Borono-2-[^18^F]fluoro-d,l-phenylalanine: a possible tracer for melanoma diagnosis with PET. Nucl Med Biol.

[11] Ishiwata K, Shiono M, Kubota K, Yoshino K, Hatazawa J, Ido T (1992). A unique in vivo assessment of 4-[^10^B]borono-l-phenylalanine in tumour tissues for boron neutron capture therapy of malignant melanoma using positron emission tomography and 4-borono-2-[^18^F]fluoro-l-phenylalanine. Melanoma Res.

[12] Kubota R, Yamada S, Ishiwata K, Tada M, Ido T, Kubota K (1993). Cellular accumulation of ^18^F-labelled boronophenylalanine depending on DNA synthesis and melanin incorporation: a double-tracer microautoradiographic study of B16 melanomas in vivo. Br J Cancer.

[13] Imahori Y, Ueda S, Ohmori Y, Yoshino E, Ono K, Kobayashi T, Mishima Y (1996). A basic concept for PET-BNCT system. Cancer neutron capture therapy.

[14] Ueda S, Imahori Y, Ohmori Y, Yoshino E, Ono K, Kobayashi T, Mishima Y (1996). Positron emission tomography and boron neutron capture therapy system to the patient with malignant brain tumor: the first clinical trial using ^10^B-BPA. Cancer neutron capture therapy.

[15] Imahori Y, Ueda S, Ohmori Y, Sakae K, Kusuki T, Kobayashi T (1998). Positron emission tomography-based boron neutron capture therapy using boronophenylalanine for high-grade gliomas: part I. Clin Cancer Res.

[16] Mishima Y, Imahori Y, Honda C, Hiratsuka J, Ueda S, Ido T (1997). In vivo diagnosis of human malignant melanoma with positron emission tomography using specific melanoma-seeking ^18^F-DOPA analogue. J Neurooncol.

[17] Imahori Y, Ueda S, Ohmori Y, Kusuki T, Ono K, Fujii R (1998). Fluorine-18-labeled fluoroboronophenylalanine PET in patients with glioma. J Nucl Med.

[18] Imahori Y, Ueda S, Ohmori Y, Sakae K, Kusuki T, Kobayashi T (1998). Positron emission tomography-based boron neutron capture therapy using boronophenylalanine for high-grade gliomas: part II. Clin Cancer Res.

[19] Kabalka GW, Smith GT, Dyke JP, Reid WS, Longford CPD, Roberts TG (1997). Evaluation of fluorine-18-BPA-fructose for boron neutron capture treatment planning. J Nucl Med.

[20] Menichetti L, Cionini L, Sauerwein WA, Altieri S, Solin O, Minn H (2009). Positron emission tomography and [^18^F]BPA: a perspective application to assess tumour extraction of boron in BNCT. Appl Radiat Isot.

[21] Nariai T, Ishiwata K, Sauerwein WAG, Wittig A, Moss R, Nakagawa Y (2012). Analysis and imaging: PET. Neutron capture therapy, principles and applications.

[22] Evangelista L, Jori G, Martini D, Sotti G (2013). Boron neutron capture therapy and ^18^F-labelled borophenylalanine positron emission tomography: a critical and clinical overview of the literature. Appl Radiat Isot.

[23] Miyatake S, Kawabata S, Hiramatsu R, Kuroiwa T, Suzuki M, Kondo N (2016). Boron neutron capture therapy for malignant brain tumors. Neurol Med Chir (Tokyo).

[24] Blue T, Yanch J (2003). Accelerator-based epithermal neutron sources for boron neutron capture therapy of brain tumors. J Neurooncol.

[25] Kreiner AJ, Bergueiro J, Cartelli D, Baldo M, Castell W, Asoia JG, Padulo J, Suárez Sandín JC, Igarzabal M, Erhardt J, Mercuri D, Valda AA, Minsky DM, Debray ME, Somacal HR, Capoulat ME, Herrera MS, Del Grosso MF, Gagetti L, Anzorena MS, Canepa N, Real N, Gun M, Tacca H (2016). Present status of accelerator-based BNCT. Rep Pract Oncol Radiother.

[26] Tanaka H, Sakurai Y, Suzuki M, Masunaga S, Kinashi Y, Kashino G (2009). Characteristics comparison between a cyclotron-based neutron source and KUR-HWNIF for boron neutron capture therapy. Nucl Instrum Methods Phys Res B.

[27] Tanaka H, Sakurai Y, Suzuki M, Masunaga S, Mitsumoto T, Fujita K (2011). Experimental verification of beam characteristics for cyclotron-based epithermal neutron source (C-BENS). Appl Radiat Isot.

[28] Wang HE, Liao AH, Deng WP, Chang PF, Chen JC, Chen FD (2004). Evaluation of 4-borono-2-^18^F-fluoro-l-phenylalanine-fructose as a probe for boron neutron capture therapy in a glioma bearing rat model. J Nucl Med.

[29] Hanaoka K, Watabe T, Naka S, Kanai Y, Ikeda H, Horitsugi G (2014). FBPA PET in boron neutron capture therapy for cancer: prediction of ^10^B concentration in the tumor and normal tissue in a rat xenograft model. EJNMMI Res.

[30] Ishiwata K, Ebinuma R, Watanabe C, Hayashi K, Toyohara J (2018). Reliable radiosynthesis of 4-[^10^B]borono-2-[^18^F]fluoro-l-phenylalanine with quality assurance for boron neutron capture therapy-oriented diagnosis. Ann Nucl Med.

[31] Nickles RJ, Daube ME, Ruth TJ (1984). An ^18^O_2_ target for the production of [^18^F]F_2_. Int J Appl Radiat Isot.

[32] Bergman J, Solin O (1997). Fluorine-18-labeled fluorine gas for synthesis of tracer molecules. Nucl Med Biol.

[33] Mairinger S, Stanek J, Wanek T, Langer O, Kuntner C (2015). Automated electrophilic radiosynthesis of [^18^F]FBPA using a modified nucleophilic GE TRACERlab FX_FDG_. Appl Radiat Isot.

[34] Vähätalo JK, Eskola O, Bergman J, Forsback S, Lehikoinen P, Jääskeläinen J (2002). Synthesis of 4-dihydroxyboryl-2-[^18^F]fluorophenylalanine with relatively high-specific activity. J Label Compd Radiopharm.

[35] Havu-Aurén K, Kiiski J, Lehtiö K, Eskola O, Kulvik M, Vuorinen V (2007). Uptake of 4-borono-2-[^18^F]fluoro-l-phenylalanine in sporadic and neurofibromatosis 2-related schwannoma and meningioma studied with PET. Eur J Nucl Med Mol Imaging.

[36] Hamacher K, Coenen HH, Stöcklin G (1986). Efficient stereospecific synthesis of no-carrier-added [^18^F]-fluoro-2-deoxy-d-glucose using aminopolyether supported nucleophilic substitution. J Nucl Med.

[37] Wingelhofer B, Kreis K, Mairinger S, Muchitsch V, Stanek J, Wanek T (2016). Preloading with l-BPA, l-tyrosine and l-DOPA enhances the uptake of [^18^F]FBPA in human and mouse tumour cell lines. Appl Radiat Isot.

[38] Coderre JA, Glass JD, Fairchild RG, Roy U, Cohen S, Fand I (1987). Selective targeting of boronophenylalanine to melanoma in BALB/c mice for neutron capture therapy. Cancer Res.

[39] Kanazawa M, Nishiyama S, Hashimoto F, Kakiuchi T, Tsukada H (2018). Evaluation of d-isomers of 4-borono-2-^18^F-fluoro-phenylalanine and *O*-^11^C-methyltyrosine as brain tumor imaging agents: a comparative PET study with their l-isomers in rat brain glioma. EJNMMI Res.

[40] Lin YC, Hwang JJ, Wang SJ, Yang BH, Chang CW, Hsiao MC (2012). Macro- and microdistributions of boron drug for boron neutron capture therapy in an animal model. Anticaner Res.

[41] Chen JC, Chang SM, Hsu FY, Wang HE, Liu RS (2004). MicroPET-based pharmacokinetic analysis of the radiolabeled boron compound [^18^F]FBPA-F in rats with F98 glioma. Appl Radiat Isot.

[42] Watabe T, Ikeda H, Nagamori S, Wiriyasermkul P, Tanaka Y, Naka S (2017). ^18^F-FBPA as a tumor-specific probe of L-type amino acid transporter 1 (LAT1): a comparison study with ^18^F-FDG and ^11^C-methionine PET. Eur J Nucl Med Mol Imaging.

[43] Yoshimoto M, Kurihara H, Honda N, Kawai K, Ohe K, Fujii H (2013). Predominant contribution of L-type amino acid transporter to 4-borono-2-^18^F-fluoro-phenylalanine uptake in human glioblastoma cells. Nucl Med Biol.

[44] Porcari P, Capuani S, D’Amore E, Lecce M, La Bella A, Fasano F (2008). In vivo ^19^F MRI and ^19^F MRS of ^19^F-labelled boronophenylalanine-fructose complex on a C6 rat glioma model to optimize boron neutron capture therapy (BNCT). Phys Med Biol.

[45] Geninatti-Crich S, Deagostino A, Toppino A, Alberti D, Venturello P, Aime S (2012). Boronated compounds for imaging guided BNCT applications. Anticancer Agents Med Chem.

[46] Bradshaw KM, Schweizer MP, Glover GH, Hadley JR, Tippets R, Tang PP (1995). BSH distributions in the canine head and a human patient using ^11^B MRI. Mag Reson Med.

[47] Kabalka GW, Tang C, Bendel P (1997). The role of boron MRI in boron neutron capture therapy. J Neurooncol.

[48] Bendel P, Koudinova N, Salomon Y (2001). In vivo imaging of the neutron capture therapy agent BSH in mice using ^10^B MRI. Magn Reson Med.

[49] Wu CY, Chan PC, Chou LS, Chang CW, Yang FY, Liu RS (2014). Pulsed-focused ultrasound enhances boron drug accumulation in a human head and neck cancer xenograft-bearing mouse model. Mol Imaging Biol.

[50] Bailey SF, Kabalka GW, Fuhr JE (1997). In vitro effects of boron-containing compounds upon glioblastoma cells. Proc Soc Exp Biol Med.

[51] Chandra S, Kabalka GW, Lorey IIDR, Smith DR, Coderre JA (2002). Imaging of fluorine and boron from fluorinated boronophenylalanine in the same cell at organelle resolution by correlative ion microscopy and confocal laser scanning microscopy. Clin Cancer Res.

[52] Wang HE, Wu SY, Chang CW, Liu RS, Hwang LC, Lee TW (2005). Evaluation of F-18-labeled amino acid derivatives and [^18^F]FDG as PET probes in a brain tumor-bearing animal model. Nucl Med Biol.

[53] Wittig A, Sauerwein WA, Coderre JA (2000). Mechanisms of transport of *p*-borono-phenylalanine through the cell membrane in vitro. Radiat Res.

[54] Detta A, Cruickshank GS (2009). l-Amino acid transporter-1 and boronophenylalanine-based boron neutron capture therapy of human brain tumors. Cancer Res.

[55] Wongthai P, Hagiwara K, Miyoshi Y, Wiriyasermkul P, Wei L, Ohgaki R (2015). Boronophenylalanine, a boron delivery agent for boron neutron capture therapy, is transported by ATB^0,+^, LAT1 and LAT2. Cancer Sci.

[56] Coenen HH, Kling P, Stöcklin G (1989). Cerebral metabolism of l-[2-^18^F]fluorotyrosine, a new PET tracer for protein synthesis. J Nucl Med.

[57] Bendel P, Wittig A, Basilico F, Mauri PL, Sauerwein W (2010). Metabolism of borono-phenylalanine–fructose complex (BPA-fr) and borocaptate sodium (BSH) in cancer patients—results from EORTC trial 11001. J Pharm Biomed Anal.

[58] Grunewald C, Sauberer M, Filip T, Wanek T, Stanek J, Mairinger S (2017). On the applicability of [^18^F]FBPA to predict l-BPA concentration after amino acid preloading in HuH-7 liver tumor model and the implication for liver boron neutron capture therapy. Nucl Med Biol.

[59] Isohashi K, Shimosegawa E, Naka S, Kanai Y, Horitsugi G, Mochida I (2016). Comparison of the image-derived radioactivity and blood-sample radioactivity for estimating the clinical indicators of the efficacy of boron neutron capture therapy (BNCT): 4-borono-2-^18^F-fluorophenylalanine (FBPA) PET study. EJNMMI Res.

[60] Watabe T, Hanaoka K, Naka S, Kanai Y, Ikeda H, Aoki M (2017). Practical calculation method to estimate the absolute boron concentration in tissues using ^18^F-FBPA PET. Ann Nucl Med.

[61] Watanabe T, Hattori Y, Ohta Y, Ishimura M, Nakagawa Y, Sanada Y (2016). Comparison of the pharmacokinetics between l-BPA and l-FBPA using the same administration dose and protocol: a validation study for the theranostic approach using [^18^F]-l-FBPA positron emission tomography in boron neutron capture therapy. BMC Cancer.

[62] Chadha M, Capala J, Coderre JA, Elowitz EH, Iwai J, Joel DD (1998). Boron neutron-capture therapy (BNCT) for glioblastoma multiforme (GBM) using the epithermal neutron beam at the Brookhaven National Laboratory. Int J Radiat Oncol Biol Phys.

[63] Yoshimoto M, Honda N, Kurihara H, Hiroi K, Nakamura S, Ito M (2018). Non-invasive estimation of ^10^B-4-borono-l-phenylalanine derived boron concentration in tumors by PET using 4-borono-2-^18^F-fluoro-phenylalanine. Cancer Sci.

[64] Papaspyrou M, Feinendegen EL, Muüller-Gärtner HW (1994). Preloading with l-tyrosine increases the uptake of boronophenylalanine in mouse melanoma cells. Cancer Res.

[65] Capuani S, Gili T, Bozzali M, Russo S, Porcari P, Cametti C (2008). l-DOPA preloading increases the uptake of borophenylalanine in C6 glioma rat model: a new strategy to improve BNCT efficacy. Int J Radiat Oncol Biol Phys.

[66] Yang W, Barth RF, Huo T, Kabalka GW, Shaikh AL, Haider SA (2014). Effects of l-DOPA pre-loading on the uptake of boronophenylalanine using the F98 glioma and B16 melanoma models. Appl Radiat Isot.

[67] Porcari P, Capuani S, D’Amore E, Lecce M, La Bella A, Fasano F (2009). In vivo ^19^F MR imaging and spectroscopy for the BNCT optimization. Appl Radiat Isot.

[68] Barth RF, Yang W, Rotaru JH, Moeschberger ML, Joel DD, Nawrocky MM (1997). Boron neutron capture therapy of brain tumors: enhanced survival following intracarotid injection of either sodium borocaptate or boronophenylalanine with or without blood–brain barrier disruption. Cancer Res.

[69] Barth RF, Yang W, Bartus RT, Moeschberger ML, Goodman JH (1999). Enhanced delivery of boronophenylalanine for boron neutron capture therapy of brain tumors using the bradykinin analog cereport (receptor-mediated permeabilizer-7). Neurosurgery.

[70] Barth RF, Yang W, Bartus RT, Rotaru JH, Ferketich AK, Moeschberger ML (2002). Neutron capture therapy of intracerebral melanoma: enhanced survival and cure after blood–brain barrier opening to improve delivery of boronophenylalanine. Int J Radiat Oncol Biol Phys.

[71] Hsieh CH, Chen YF, Chen FD, Hwang JJ, Chen JC, Liu RS (2005). Evaluation of pharmacokinetics of 4-borono-2-^18^F-fluoro-l-phenylalanine for boron neutron capture therapy in a glioma-bearing rat model with hyperosmolar blood–brain barrier disruption. J Nucl Med.

[72] Yang FY, Chang WY, Li JJ, Wang HE, Chen JC, Chang CW (2014). Pharmacokinetic analysis and uptake of ^18^F-FBPA-Fr after ultrasound-induced blood–brain barrier disruption for potential enhancement of boron delivery for neutron capture therapy. J Nucl Med.

[73] Sakata M, Oda K, Toyohara J, Ishii K, Nariai T, Ishiwata K (2013). Direct comparison of radiation dosimetry of six PET tracers using human whole-body imaging and murine biodistribution studies. Ann Nucl Med.

[74] Kono Y, Kurihara H, Kawamoto H, Yasui N, Honda N, Igaki H (2017). Radiation absorbed dose estimates for ^18^F-BPA PET. Acta Radiol.

[75] Pöpperl G, Kreth FW, Mehrkens JH, Herms J, Seelos K, Koch W (2007). FET PET for the evaluation of untreated gliomas: correlation of FET uptake and uptake kinetics with tumour grading. Eur J Nucl Med Mol Imaging.

[76] Kratochwil C, Combs SE, Leotta K, Afshar-Oromieh A, Rieken S, Debus J (2014). Intra-individual comparison of ^18^F-FET and ^18^F-DOPA in PET imaging of recurrent brain tumors. Neuro Oncol.

[77] Koopman T, Verburg N, Schuit RC, Pouwels PJW, Wesseling P, Windhorst AD (2018). Quantification of *O*-(2-[^18^F]fluoroethyl)-l-tyrosine kinetics in glioma. EJNMMI Res.

[78] Takahashi Y, Imahori Y, Mineura K (2003). Prognostic and therapeutic indicator of fluoroboronophenylalanine positron emission tomography in patients with gliomas. Clin Cancer Res.

[79] Nichols T, Kabalka GW, Miller LF, Khan MK, Smith GT (2002). Improved treatment planning for boron neutron capture therapy for glioblastoma multiforme using fluorine-18 labeled boronophenylalanine and positron emission tomography. Med Phys.

[80] Kabalka GW, Nichols TL, Smith GT, Miller LF, Khan MK, Busse PM (2003). The use of positron emission tomography to develop boron neutron capture therapy treatment plans for metastatic malignant melanoma. J Neurooncol.

[81] Koivunoro H, Hippeläinen E, Auterinen I, Kankaanranta L, Kulvik M, Laakso J (2015). Biokinetic analysis of tissue boron (^10^B) concentrations of glioma patients treated with BNCT in Finland. Appl Radiat Isot.

[82] Soloway AH, Tjarks W, Barnum BA, Rong FG, Barth RF, Codogni IM (1998). The chemistry of neutron capture therapy. Chem Rev.

[83] Barth RF, Soloway AH (1997). Boron neutron capture therapy of brain tumors—current status and future prospects. J Neurooncol.

[84] Nariai T, Ishiwata K, Kimura Y, Inaji M, Momose T, Yamamoto T (2009). PET pharmacokinetic analysis to estimate boron concentration in tumor and brain as a guide to plan BNCT for malignant cerebral glioma. Appl Radiat Isot.

[85] Morita T, Kurihara H, Hiroi K, Honda N, Igaki H, Hatazawa J (2018). Dynamic changes in ^18^F-borono-l-phenylalanine uptake in unresectable, advanced, or recurrent squamous cell carcinoma of the head and neck and malignant melanoma during boron neutron capture therapy patient selection. Radiat Oncol.

[86] Ariyoshi Y, Shimahara M, Kimura Y, Ito Y, Shimahara T, Miyatake S (2011). Fluorine-18-labeled boronophenylalanine positron emission tomography for oral cancers: Qualitative and quantitative analyses of malignant tumors and normal structures in oral and maxillofacial regions. Oncol Lett.

[87] Suzuki M, Kato I, Aihara T, Hiratsuka J, Yoshimura K, Niimi M (2014). Boron neutron capture therapy outcomes for advanced or recurrent head and neck cancer. J Radiat Res.

[88] Shimosegawa E, Isohashi K, Naka S, Horitsugi G, Hatazawa J (2016). Assessment of ^10^B concentration in boron neutron capture therapy: potential of image-guided therapy using ^18^FBPA PET. Ann Nucl Med.

[89] Nedunchezhian K, Aswath N, Thiruppathy M, Thirugnanamurthy S (2016). Boron neutron capture therapy—a literature review. J Clin Diagn Res.

[90] Kankaanranta L, Seppälä T, Koivunoro H, Saarilahti K, Atula T, Collan J (2012). Boron neutron capture therapy in the treatment of locally recurred head-and-neck cancer: final analysis of a phase I/II trial. Int J Radiat Oncol Biol Phys.

[91] Wang LW, Wang SJ, Chu PY, Ho CY, Jiang SH, Liu YWH (2011). BNCT for locally recurrent head and neck cancer: preliminary clinical experience from a phase I/II trial at Tsing Hua open-pool reactor. Appl Radiat Isot.

[92] Wang LW, Liu YWH, Chou FI, Jiang D-H (2018). Clinical trials for treating recurrent head and neck cancer with boron neutron capture therapy using the Tsing–Hua open pool reactor. Cancer Commun.

[93] Aiyama H, Nakai K, Yamamoto T, Nariai T, Kumada H, Ishikawa E (2011). A clinical trial protocol for second line treatment of malignant brain tumors with BNCT at University of Tsukuba. Appl Radiat Isot.

[94] Miyatake S, Furuse M, Kawabata S, Maruyama T, Kumabe T, Kuroiwa T (2013). Bevacizumab treatment of symptomatic pseudoprogression after boron neutron capture therapy for recurrent malignant gliomas. Report of 2 cases. Neuro Oncol.

[95] Kato I, Fujita Y, Maruhashi A, Kumada H, Ohmae M, Kirihata M (2009). Effectiveness of boron neutron capture therapy for recurrent head and neck malignancies. Appl Radiat Isot.

[96] Yamamoto T, Nakai K, Nariai T, Kumada H, Okumura T, Mizumoto M (2011). The status of Tsukuba BNCT trial: BPA-based boron neutron capture therapy combined with X-ray irradiation. Appl Radiat Isot.

[97] Watanabe Y, Kurihara H, Itami J, Sasaki R, Arai Y, Sugimura K (2017). Relationship between the uptake of ^18^F-borono-l-phenylalanine and l-[methyl-^11^C]methionine in head and neck tumors and normal organs. Radiat Oncol.

[98] Tani H, Kurihara H, Hiroi K, Honda N, Yoshimoto M, Kono Y (2014). Correlation of ^18^F-BPA and ^18^F-FDG uptake in head and neck cancers. Radiother Oncol.

[99] Kobayashi K, Kurihara H, Watanabe Y, Murakami N, Inaba K, Nakamura S (2016). In vivo spatial correlation of ^18^F-BPA and ^18^F-FDG uptake in head and neck cancer. Appl Radiat Isot.

[100] Miyashita M, Miyatake S, Imahori Y, Yokoyama K, Kawabata S, Kajimoto Y (2008). Evaluation of fluoride-labeled boronophenylalanine-PET imaging for the study of radiation effects in patients with glioblastomas. J Neurooncol.

[101] Miyatake S, Kawabata S, Nonoguchi N, Yokoyama K, Kuroiwa T, Ono K (2009). Pseudoprogression in boron neutron capture therapy for malignant gliomas and meningiomas. Neuro Oncol.

[102] Kawabata S, Miyatake S, Kuroiwa T, Yokoyama K, Doi A, Iida K (2009). Boron neutron capture therapy for newly diagnosed glioblastoma. J Radiat Res.

[103] Miyatake S, Kawabata S, Hiramatsu R, Furuse M, Kuroiwa T, Suzuki M (2014). Boron neutron capture therapy with bevacizumab may prolong the survival of recurrent malignant glioma patients: four cases. Radiat Oncol.

[104] Tsuyuguchi N, Sunada I, Iwai Y, Yamanaka K, Tanaka K, Takami T (2003). Methionine positron emission tomography of recurrent metastatic brain tumor and radiation necrosis after stereotactic radiosurgery: is a differential diagnosis possible?. J Neurosurg.

[105] Beshr R, Isohashi K, Watabe T, Naka S, Horitsugi G, Romanov V (2018). Preliminary feasibility study on differential diagnosis between radiation-induced cerebral necrosis and recurrent brain tumor by means of [^18^F]fluoro-borono-phenylalanine PET/CT. Ann Nucl Med.

